# Glaucoma

**Published:** 1893-11

**Authors:** A. Bethune Patterson

**Affiliations:** Atlanta, Ga., Late Assistant to the Royal London Ophthalmic Hospital and Golden Square Throat Hospital, London, England


### GLAUCOMA.

BY A. BETHUNE PATTERSON, M. D., ATLANTA, GA.,

Late Assistant to the Royal London Ophthalmic Hospital and Golden Square Throat Hospital, London, England.

There is a growing tendency among specialists to confine their contributions to journals devoted to their special branch of medicine, and as a fact this class of periodicals are on the increase; while this course may be be considered commendable, as tending to the production of original matter, yet I am strongly convinced that a rigid adherence to this modern tendency is calculated to work an injury to general medicine, in as much as general practitioners are deprived of the observations and experiences of this class of their colleagues, of which they often feel the need and which they are justly entitled to, for they are not always in a position to confer and consult with specialist upon matters that are daily arising, and which call for special training and experience. This is especially the case in many insidious diseases, intricate in diagnoses. Having recently had cases under observation which illustrates the point in question, and which to some extent has inspired a desire to lend a helping hand in aid of our busy practioners, and with that view I have selected as a subject for consideration the disease known as glaucoma.

I regret to say that I have nothing original to offer, either in the pathology, etiology or treatment of this formidable disease, a disease so apt to be overlooked, moving slothfully and surely on until advanced to the extent of hopelessness, though frequently when early recognized, the fatal day can be postponed, the age made comfortable and sight prolonged, if not cured upon the spot.

The nomenclature of the disease under consideration deserves revision at the hands of the book-makers, the word glaucoma is derived from the Greek, meaning sea-green, and is expressive only of the appearance in the latter stage of the dreaded malady. The etiology of glaucoma is obscure, there are several theories advanced which do not deserve consideration here. It is to be remembered, however, that it is a disease of advanced life. Our knowledge of the pathology is in keeping with the cause. The symptoms are quite enough for the purpose of this paper, and only propose to deal with a few of them.

There are quite a number of subjective and objective symptoms, though rarely all are ever seen in an individual case. Authors place great stress upon "tension," the eye ball loses its elasticity and does not respond to slight pressure from the finger, (palpation) the scale of tension varies from slight unyieldingness to "stony-hardness." It requires considerable experience to be able to detect a slight increase in tension and in the early stage cannot be relied upon in making a diagnosis, later it becomes an infallible evidence. Pain, neuralgic in character, varying from a slight twinge to a severe paroxysm and generally nocturnal at the outset. Pain in the eye and head in elderly people should always excite the suspicion of the practitioner, these early attacks are apt to be overlooked in malarial districts, as pain in head, temples and eyes generally accompanies malarial toxæmia. In acute glaucoma the pain is unbearable, and is more characteristic, while in sub-acute and chronic stage, pain is not so marked a feature, scarcely noticeable or sufficient to call for advice.

The next symptom to attract the attention of the patient, is the failing in sight, especially is this the case in presbyopic patients, who have to make frequent changes in their glasses. In chronic glaucoma this peculiarity is quite often the only symptom, yet deterioation in central vision is not very rapid, while there is generally a contraction of the field of vision more noticeable on the nasal side. It is not unusual to see these mild cases suddenly converted into the acute or sub-acute attacks with a rapid loss of sight. A deceptive feature often observed in sub-acute glaucoma, is the recovery of the sight, which was partially lost during the attack, while in acute attacks sight is noticed to be worse on recovery from the periodical attacks, and it is often the case that vision becomes almost lost from first attack. Dilated pupils, shallow anterior chamber, congestion of episcleral vessels, with pain in head and temples with diminution of sight combine to make up sub-acute attack, which, as the name implies, only differs from the acute in severity of symptoms.

In chronic glaucoma the tell-tale symptom may simply be imperfect sight, which frequently, however, does not attract much attention from patient, as the cloudiness passes off in a few days to again appear in a few weeks or months. After a few such attacks the patient becomes distressed at the permanency of the impaired sight. Inflammation and pain are not characteristic of this stage of the disease. though frequently play a part in the retrograde conditions, colored lights, "rain bows," "halos" seen around lamp and candle flames are significant symptoms in this disease. The inflammatory conditions of conjunctiva in acute glaucoma are apt to be mistaken for acute ophthalmia. Inspection of pupil would assist in dispelling the doubt, as in the latter disease the pupil is contracted, while in glaucoma it would be dilated, more or less sluggish, and quite often inactive. This condition of the pupil with shallow anterior chamber is characteristic, though cases are frequently observed in the incipient stage where dilatation is not presented, and that too, where pain is a marked feature, though subsequently become widely dilated.

While the prognosis is always considered grave, it should be borne in mind that successful management depends upon early recognition. The family physician is invariably first consulted, and upon his recognition of the disease, and the advice given will determine the future condition of sight and happiness of the patient. In considering treatment, three divisions are to be made. Namely, prophylactic, myotic and surgical. As to the first, little can be said owing to our lack of knowledge as to cause. Recognizing the disease to be one of advanced life, we naturally look to the presbyopic conditions as playing a part in the etiological role. We are all familiar with the pain in eyes and head and the conjestion of the conjunctival vessels so common in eye strain due to astigmatism, hypermetropia and presbyopia, and when accompanied by a run-down condition of health, a gouty and rheumatic dyscrasia seem to be quite enough to bring about the degenerated condition of the eye tunics. Every effort should be used to eliminate eye strain, astigmatism, hypermetropia, and presbyopia, the degree should be estimated and accurately corrected with properly adjusted glasses. I wish to accentuate the phrase "properly adjusted," care should be exercised in selecting frames, as the correct position of the glass must depend upon a correctly adjusted frame, patients should look through the center of this glass, if above or below this point, or out of the corners of the glass, eye strain will surely result. Glasses are remedies and should never be selected by patient, and under no circumstances should this important piece of scientific work be entrusted to the optician. Treatment by myotics consist in the instillation of eserene and pilocarpine, half grain of the former, and four grains of the latter to an ounce of water, a few drops instilled into eyes every three or four hours, the pupil is thus contracted to a point, drawn away from the angle of anterior chamber, the base of iris thus facilitating free drainage. Iredictomy is the surgical method of treatment, and should be performed as early as practicable in acute glaucoma. In the chronic stage iridectomy is not as satisfactory, it is here that the myotic treatment proves most satisfactory.

